# Booster Vaccination With GVGH *Shigella sonnei* 1790GAHB GMMA Vaccine Compared to Single Vaccination in Unvaccinated Healthy European Adults: Results From a Phase 1 Clinical Trial

**DOI:** 10.3389/fimmu.2019.00335

**Published:** 2019-03-08

**Authors:** Odile Launay, Augustin G. W. Ndiaye, Valentino Conti, Pierre Loulergue, Antonella Silvia Sciré, Anais Maugard Landre, Pietro Ferruzzi, Naouel Nedjaai, Lena Dorothee Schütte, Joachim Auerbach, Elisa Marchetti, Allan Saul, Laura B. Martin, Audino Podda

**Affiliations:** ^1^Université Paris Descartes, Sorbonne Paris Cité, Paris, France; ^2^Inserm CIC 1417, F-CRIN I-REIVAC, Paris, France; ^3^Assistance Publique Hôpitaux de Paris, CIC Cochin-Pasteur, Paris, France; ^4^GSK Vaccines Institute for Global Health, Siena, Italy; ^5^GSK, Marburg, Germany

**Keywords:** *Shigella sonnei*, 1790GAHB, GMMA (generalized modules for membrane antigen), booster response, antibody persistence, safety

## Abstract

The investigational *Shigella sonnei* vaccine (1790GAHB) based on GMMA (generalized modules for membrane antigens) is immunogenic, with an acceptable safety profile in adults. However, pre-vaccination anti-*S. sonnei* lipopolysaccharide (LPS) antibody levels seemed to impact vaccine-related immune responses. This phase 1, open-label, non-randomized extension study (ClinicalTrials.gov: NCT03089879) evaluated immunogenicity of a 1790GAHB booster dose in seven adults with undetectable antibodies prior to priming with three 1790GAHB vaccinations 2–3 years earlier (boosted group), compared to one dose in 28 vaccine-naïve individuals (vaccine-naïve group). Anti-*S. sonnei* LPS serum IgG geometric mean concentrations and seroresponse (increase of ≥25 EU or ≥50% from baseline antibody ≤ 50 EU and ≥50 EU, respectively) rates were calculated at vaccination (day [D]1), D8, D15, D29, D85. Safety was assessed. Geometric mean concentrations at D8 were 168 EU (boosted group) and 32 EU (vaccine-naïve group). Response peaked at D15 (883 EU) and D29 (100 EU) for the boosted and vaccine-naïve groups. Seroresponse rates at D8 were 86% (boosted group) and 24% (vaccine-naïve group) and increased at subsequent time points. Across both groups, pain (local) and fatigue (systemic) were the most frequent solicited adverse events (AEs). Unsolicited AEs were reported by 57% of boosted and 25% of vaccine-naïve participants. No deaths, serious AEs, or AEs of special interest (except one mild neutropenia case, possibly vaccination-related) were reported. One 1790GAHB dose induced a significant booster response in previously-primed adults, regardless of priming dose, and strong immune response in vaccine-naïve individuals. Vaccination was well tolerated.

## Introduction

Diarrheal diseases continue to represent a major cause of death worldwide, with more than 1.6 million fatalities estimated in 2016 (1). Among the three pathogens causing the majority of diarrhea deaths, *Shigella* accounted for 212,438 estimated deaths in all ages and 37,034 in children under 5 years of age, the majority in low-middle income countries (2). The *Shigella* genus encompasses four species and 50 serotypes, differentiated on the basis of the variability of their O antigen (OAg), part of the lipopolysaccharide (LPS) in the outer membrane of the bacteria (3). The global epidemiology of *Shigella* is changing constantly, but recently, the single serotype of *S. sonnei* has shown a significant increase in prevalence in several parts of the world (4–8). Early identification and antibiotic treatment are key factors in the management of shigellosis (9), but *Shigella* species have developed substantial antibiotic resistance (10–13). Therefore, the development of an effective vaccine against *Shigella* remains an important unmet medical need. Several OAg-based conjugate or live-attenuated vaccines are currently under development, but no licensed *Shigella* vaccine is widely available (14–16).

The GSK Vaccines Institute for Global Health (GVGH) investigational *S. sonnei* vaccine 1790GAHB, using GMMA (generalized modules for membrane antigens) as a delivery system for O antigen (OAg), has already been shown to be highly immunogenic and to have an acceptable safety profile in European (17) and Kenyan (18) adults. In a phase 1 study conducted in 50 French adults, five different GMMA OAg/protein doses of 1790GAHB (0.059/1 μg, 0.29/5 μg, 1.5/25 μg, 2.9/50 μg or 5.9/100 μg), administered at each of three intramuscular vaccinations 1 month apart, were compared to placebo administration (17). While the antibody response observed across all vaccine groups peaked with the 1.5/25 μg dose, no substantial difference was seen in the response of participants receiving the three highest vaccine doses (1.5/25, 2.9/50 or 5.9/100 μg) (17). Moreover, *post-hoc* analyses showed that pre-existing anti-*S. sonnei* LPS antibody levels potentially impact response to vaccination. More specifically, participants with detectable antibodies at baseline had higher antibody levels following the first vaccination and a less pronounced decline of antibody levels up to 168 days post-last vaccination than those with undetectable antibody levels at baseline (17).

Long-lived antibody is desired for an effective public health vaccine, as is the ability to boost the response, either through revaccination or infection, especially in young children not previously exposed to *Shigella*. Therefore, this extension study aimed to further characterize the immunogenicity profile of the *S. sonnei* 1790GAHB vaccine in participants with undetectable pre-vaccination antibodies. The study compared a fourth vaccination, 2–3 years after the third vaccination in the parent study, to a single vaccination in vaccine-naïve adults. Based on safety and immunogenicity results obtained in the parent trial (17, 18), a dose of 1.5/25 μg OAg/protein was selected for use in the extension trial.

**Figure 1:**
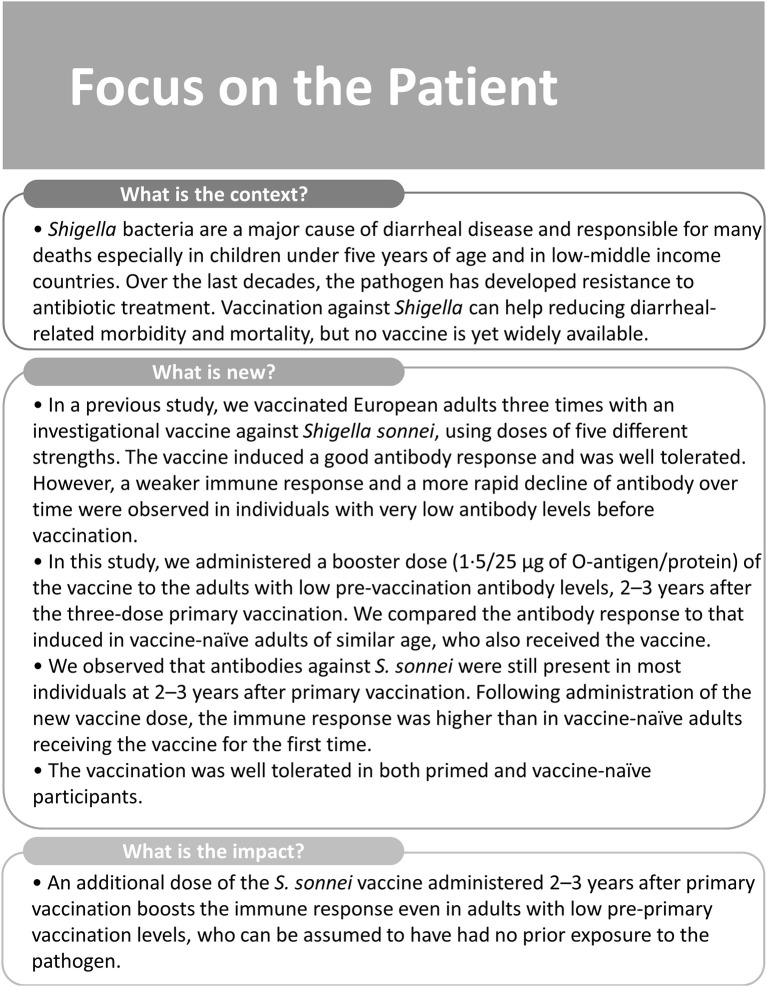
Focus on patient section.

A summary contextualizing the results and potential clinical relevance and impact of the research is displayed in the Focus on Patient Section (Figure 1), for the benefit of healthcare professionals.

## Materials and Methods

### Study Design and Participants

This open label, non-randomized, single center, phase 1, extension study (NCT03089879) was conducted in France between March and August 2017. The extension trial enrolled healthy adults from the parent study, who received three vaccinations with 1790GAHB 2–3 years earlier (boosted group) or who received placebo (17). All participants enrolled from the previous study had undetectable anti-*S. sonnei* LPS antibody levels before first vaccination in the parent study. The extension study further recruited adults with or without detectable anti-*S. sonnei* LPS antibody levels at baseline. The placebo recipients from the parent study and the newly-recruited volunteers were enrolled in the vaccine-naïve group. Individuals aged 22–50 years were eligible for participation in the extension study if they were affiliated with a social security regimen and, for women of child-bearing potential, if they had a negative urinary pregnancy test before vaccination and agreed to use acceptable birth control measures throughout the study. The full list of inclusion/exclusion criteria is provided in the Supplementary Text S1.

All participants received 0.5 mL of the *S. sonnei* 1790GAHB vaccine, by intramuscular route. The vaccine was provided as a preservative-free formulation (single vial of 0.7 mL) of *S. sonnei* 1790-GMMA (12 μg/mL measured by OAg and 200 μg/mL measured by protein content) adsorbed to Alhydrogel (0.7 mg Al^3+^/mL) in Tris-buffered saline. The 0.5 mL dose containing 1.5/25 μg of OAg/protein was obtained by dilution with Alhydrogel in Tris-buffered saline (0.7 mg Al^3+^/mL), immediately prior to vaccination. The study was conducted in accordance with Good Clinical Practice guidelines and the Declaration of Helsinki. Written informed consent was obtained from each participant prior to conducting any study-specific procedure. The protocol was approved by a National Ethic Committee (CPP EST1), assigned according to the pilot phase of the European Union Regulation No. 536/2014 for clinical trial applications in France. The study was registered at www.clinicaltrials.gov (NCT03089879) and a protocol summary is available at http://www.gsk-clinicalstudyregister.com (study ID 205905).

### Study Objectives

The primary objective was to evaluate the memory response elicited by a booster dose of 1790GAHB in primed individuals following three vaccinations with 1790GAHB in the parent study and having undetectable antibody levels prior to the primary vaccination series, as measured by enzyme-linked immunosorbent assay (ELISA). Anti-*S. sonnei* LPS serum immunoglobulin G (IgG) at seven days post-booster vaccination were compared to the administration of a single vaccine dose to vaccine-naïve participants (including placebo recipients from the parent trial and individuals enrolled in the extension trial). Secondary objectives assessed the safety and immunogenicity of 1790GAHB in all study participants, including the antibody profile of the boosted group compared to the vaccine-naïve group at baseline and 7, 14, 28, and 84 days post-vaccination, the antibody profile of the vaccine-naïve individuals with detectable antibody at baseline and at 7, 14, 28, and 84 days post-vaccination, and the persistence of anti-*S. sonnei* LPS antibody levels, at the start of the extension study, in participants primed with 1790GAHB in the parent study ~2–3 years earlier.

### Immunogenicity and Safety Assessments

Blood samples were collected as follows: ~15 mL were drawn for hematological and 25 mL for serological testing from all participants as part of the initial screening. For serological analyses, further samples of 20 mL were collected from each participant before vaccination and 7, 14, 28, and 84 days post-vaccination. At 28 days post-vaccination, an additional blood sample of 20 mL was drawn to allow the creation of a standard reference serum for subsequent studies. Additional samples of 6 mL were drawn for hematological tests at 7 and 84 dayspost-vaccination.

Serum was kept frozen below −20°C and transported to GSK (Marburg, Germany). Serologic testing was performed on one aliquot, while the others were stored for future analyses. Anti-*S. sonnei* LPS serum IgG was measured by ELISA using *S. sonnei* LPS as plate coating antigen (19). A dilution series of standard reference serum pool generated during the parent study was included on each ELISA plate. The standard reference serum was calibrated such that 1 ELISA unit (EU) equals the reciprocal of the dilution giving an optical density at 405 −490 nm of 1 in the standard assay. The ELISA detection limit varied from plate to plate, ranging between 5.5 and 7·4 EU.

Antibody responses were assessed by anti-*S. sonnei* LPS serum IgG geometric mean concentrations (GMCs) and seroresponse rates, calculated at each time point. Seroresponse was defined as a post-vaccination increase of at least 25 EU and at least 50% of anti-*S. sonnei* LPS IgG ≤ 50 EU and ≥50 EU, respectively, at baseline. A level of anti-*S. sonnei* LPS serum IgG of 121 EU was also used as a threshold for the assessment of immune response, similarly to the parent study (17). Post-vaccination levels of 121 EU were found to correspond to the median titer of 1:800 measured in the sera of convalescent individuals previously infected with *S. sonnei*, using the ELISA method by Cohen et al. (20).

After receiving 1790GAHB, participants were monitored at the study site for 4 h. Occurrence of solicited local (pain, erythema, and induration) and systemic (headache, arthralgia, chills, fatigue, malaise, myalgia, and orally-measured fever) adverse events (AEs) during the 7 days post-vaccination period were documented by the participants on diary cards. Unsolicited AEs occurring within 84 days after vaccination were collected by study staff during scheduled (at 7, 14, 28, and 84 days post-vaccination) and unscheduled clinic visits. Solicited AEs continuing beyond 7 days post-vaccination were reported as unsolicited events. Serious AEs (SAEs), AEs of special interests (AESIs; reactive arthritis and neutropenia), and AEs leading to withdrawal from the study were collected throughout the study period and assessed by the investigator as being either probably-, possibly- or not-related to vaccination.

### Statistical Analysis

No formal statistical sample size was calculated, as all analyses were descriptive. Serological assessments were carried out on the full analysis set at each time point, which included participants with at least one evaluable serum sample. For each group, GMCs were calculated with their associated two-sided 95% confidence intervals (CIs) by exponentiating the mean and 95% CIs of the logarithmically-transformed (base 10) EU. Geometric mean ratios (GMRs) and associated 95% CIs were computed for GMC at post-vaccination time points vs. pre-vaccination levels, by exponentiating the mean within-subject differences in log-transformed concentrations and the corresponding 95% CIs. For statistical analysis of ELISA data, antibody levels below the limit of detection were set to half that limit.

The number and percentage of participants with seroresponse and post-vaccination antibody level ≥121 EU for anti-*S. sonnei* LPS serum IgG was computed with 95% Clopper-Pearson CIs.

Safety analyses were performed on any solicited or unsolicited AE data collected from participants who received 1790GAHB. All solicited AEs were evaluated on a 3-grade scale as mild, moderate, or severe. The number and percentage of participants with AEs, SAEs, AESIs, new onset of chronic disease, potential immune-mediated disease, medically attended AEs, AEs leading to withdrawal, and clinically significant deviations in hematology test values were summarized.

## Results

### Demographics

**Figure 2:**
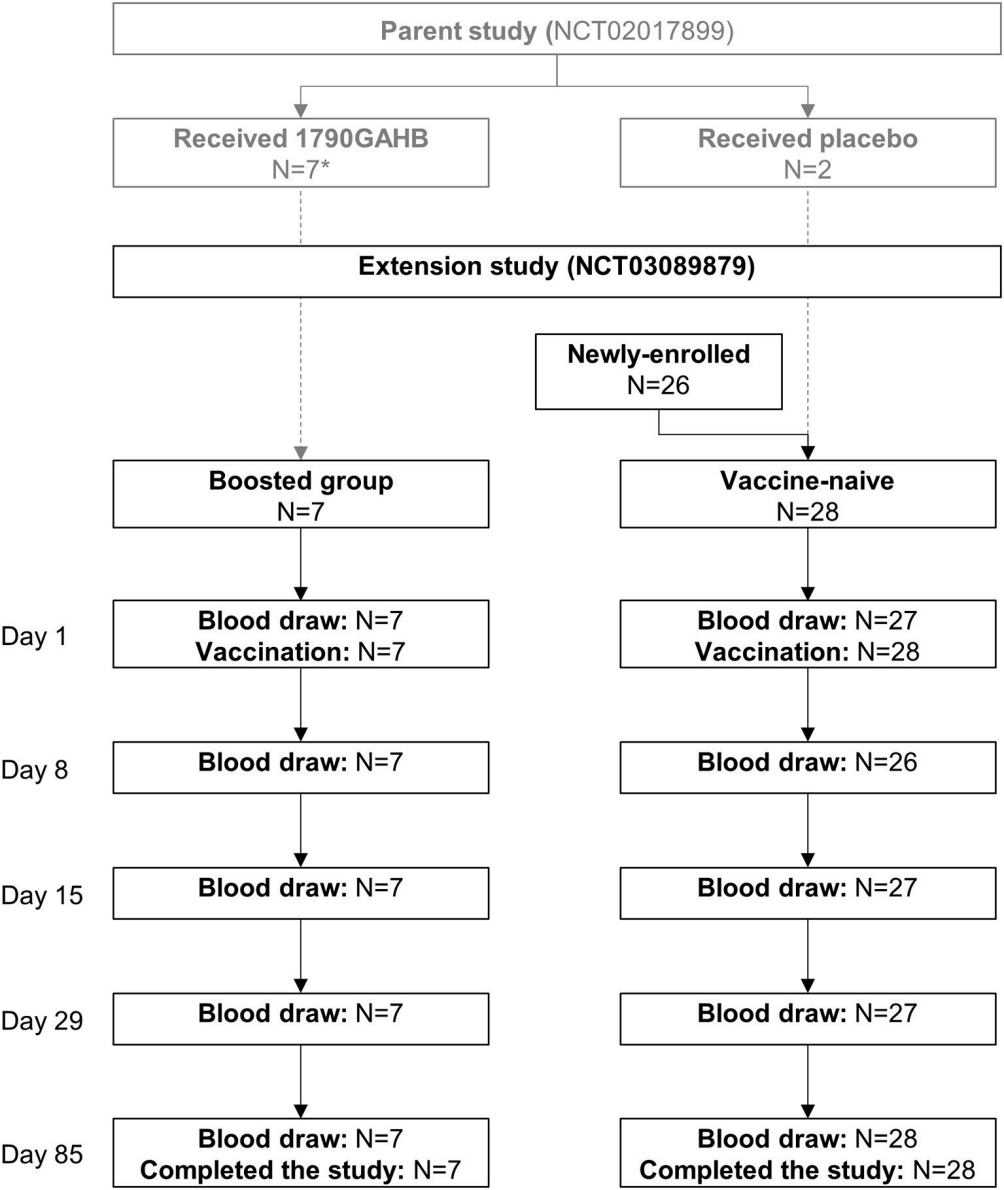
Participant flowchart. N, number of participants. *Received doses of 1790GAHB with an O-antigen/protein content of 0·059/1 μg (4 participants), 0·29/5 μg (1 participant), and 2·9/50 μg (2 participants) in the parent study.

A total of 35 adults participated in the study. Seven adults vaccinated with 1790GAHB in the parent study were re-enrolled in the boosted group. The vaccine-naïve group included two adults receiving placebo in the parent study and 26 newly-enrolled individuals. All participants received the study vaccination and completed the study (Figure 2). Demographic characteristics at enrolment in the extension trial are presented in Table 1.

**Table 1:** Participant characteristics at enrollment in extension study.

	**Boosted group (*N* = 7)**	**Vaccine-naïve group (*N* = 28)**	**Total (*N* = 35)**
Age (mean ± *SD*), years	37.7 ± 7.9	34.3 ± 8.5	34.9 ± 8.4
Male, *n* (%)	3 (42·9)	17 (60.7)	20 (57)
Race, *n* (%)			
Black	1 (14.3)	1 (3.6)	2 (6)
White	6 (85.7)	26 (92.9)	32 (91)
Other	0 (0.0)	1 (3.6)	1 (3)
Weight (mean ± *SD*), kg	63 ± 15.5	74.6 ± 11.4	72.3 ± 12.9
Height (mean ± *SD*), cm	168.6 ± 11.6	173.3 ± 10.5	172.3 ± 10.7
BMI (mean ± *SD*), kg/m^2^	21.9 ± 2.8	24.8 ± 3.0	24.2 ± 3.1

*N, number of enrolled participants in each group; SD, standard deviation; n (%), number (percentage) of participants in each category; BMI, body mass index*.

### Immunogenicity

**Figure 3:**
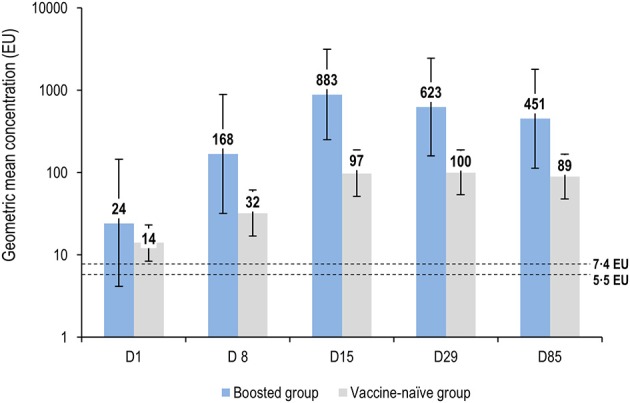
Anti-*S. sonnei* LPS IgG geometric mean concentrations by time point (full analysis set for immunogenicity). LPS, lipopolysaccharide; IgG, immunoglobulin G; EU, enzyme-linked immunosorbent assay units; D, day. Error bars represent 95% confidence intervals. Dashed lines represent the limit of detection of the enzyme-linked immunosorbent assay, which varied from plate to plate, from 5·5 to 7·4 EU.

**Figure 4:**
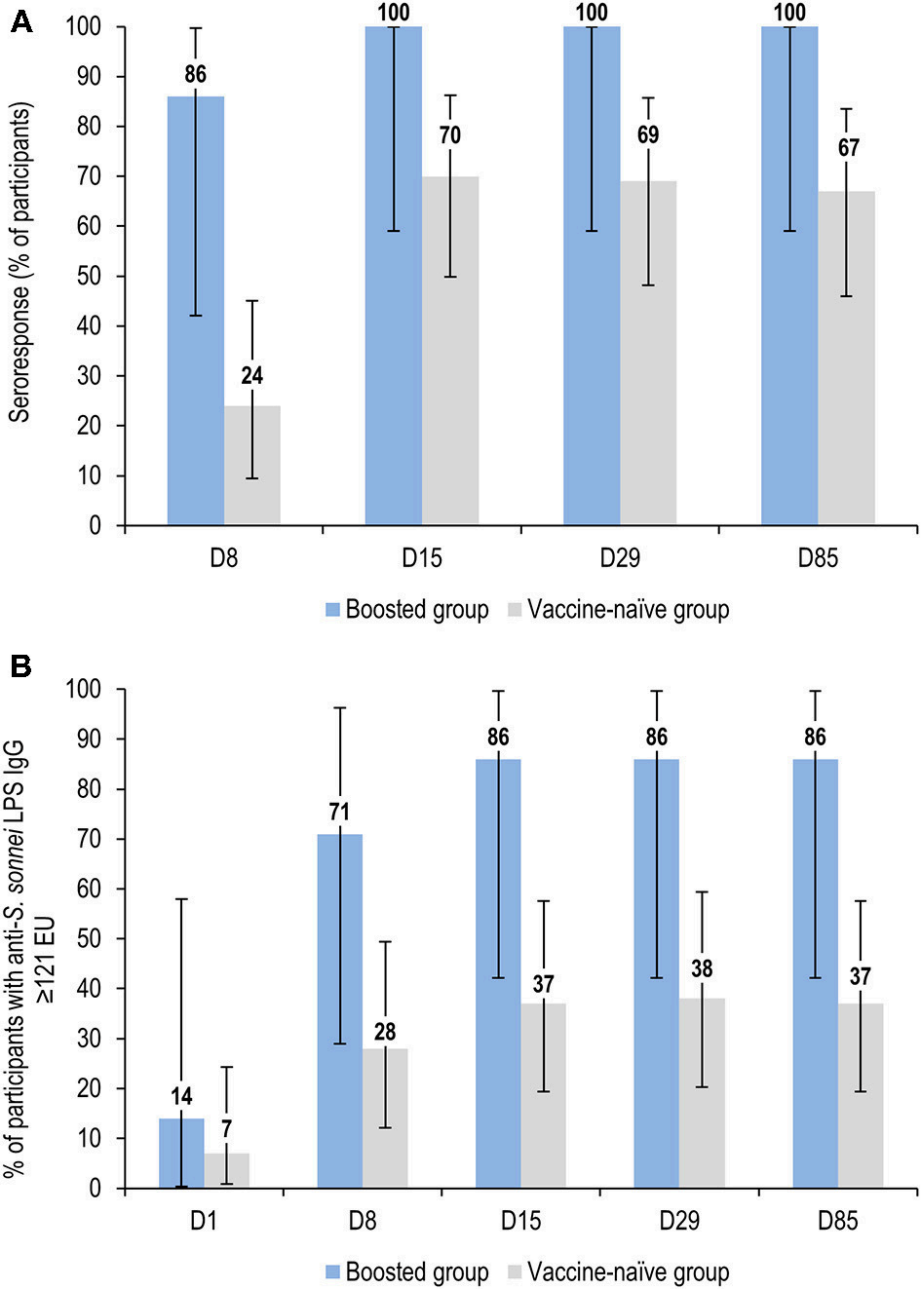
Percentage of participants with seroresponse **(A)** and anti-*S. sonnei* LPS IgG ≥121 EU **(B)** by time point (full analysis set for immunogenicity). LPS, lipopolysaccharide; IgG, immunoglobulin G; EU, enzyme-linked immunosorbent assay units; D, day. Seroresponse to vaccination was defined as an increase in the anti-*S. sonnei* LPS serum IgG level of ≥50% for participants with baseline (D1) levels >50 EU or an increase of ≥25 EU for participants with pre-vaccination (D1) levels ≤ 50 EU. Error bars represent 95% confidence intervals.

At seven days post-vaccination, anti-*S. sonnei* LPS IgG GMCs were 168 EU (95% CI: 32–889) in the boosted group compared to 32 EU (95% CI: 17–61) in the vaccine-naïve group (Figure 3; Supplementary Table 1). Seroresponse rates were 86% (95% CI: 42.1–99.64) and 24% (95% CI: 9.4–45.1) in the boosted and vaccine-naïve groups, respectively (Figure 4A; Supplementary Table 2). The percentage of individuals with anti-*S. sonnei* LPS IgG ≥121 EU was 71% (95% CI: 29.0–96.3) in the boosted group and 28% (95% CI: 12.1–49.4) in the vaccine-naïve group (Figure 4B; Supplementary Table 2).

Anti-*S. sonnei* LPS IgG GMCs increased substantially until 14 days post-vaccination in the boosted group, reaching a peak GMC of 883 EU (95% CI: 249-3126), with 100 and 86% of participants achieving seroresponse and antibody levels >121 EU, respectively. Antibody levels then declined at subsequent time points, dropping to about half the peak (GMC of 451 [95% CI: 113–1797]), at 84 days post-vaccination (Figure 4; Supplementary Tables 1, 2). Antibody responses in the vaccine-naïve group also increased following vaccination, but more slowly, and showed a broad but much lower peak, with GMCs of 97 EU (95% CI: 51–187) and 100 EU (95% CI: 54–187) at 14 and 28 days post-vaccination, respectively; then declined to 89 EU (95% CI: 48–166) at 84 days post-vaccination (Figure 4; Supplementary Table 1). At all-time points except baseline, anti-*S. sonnei* LPS IgG GMCs in the boosted group were ≥5-fold higher compared to those in vaccine-naïve participants (Figure 4; Supplementary Table 1).

The kinetics of antibody response in the vaccine-naïve group depended on the antibody levels at the time of vaccination. Those with antibody levels higher than the detection limit at baseline had antibody kinetics that more closely resembled the boosted group, albeit with a much lower peak in antibody levels. GMCs peaked at 14 days post-vaccination (137 [95% CI: 65–289]), subsequently falling to a GMC of 110 (95%CI: 51–233) at 84 post-vaccination with 1790GAHB. By contrast, adults in the vaccine-naïve group with no detectable antibody at baseline had a slower and smaller raise in antibody; GMCs were 43 (95% CI: 11–175) and 55 (95% CI: 17–174) at 14 and 28 days post-vaccination, with only a small further decrease to a GMC value of 54 (14–208) at 84 days post-vaccination.

**Figure 5:**
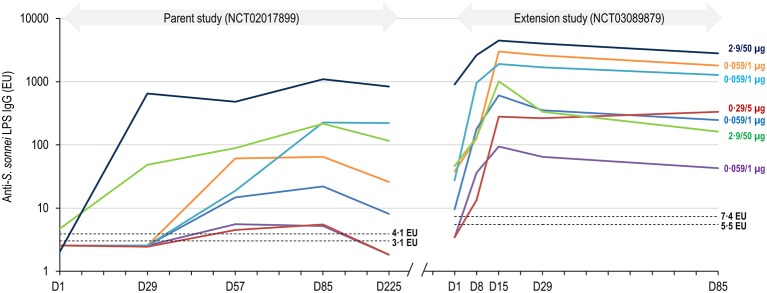
Individual anti-S. sonnei LPS IgG responses of participants in the boosted group throughout the parent and extension studies. LPS, lipopolysaccharide; IgG, immunoglobulin G; EU, enzyme-linked immunosorbent assay units; D, day. The interval from D225 in the parent study and D1 in the extension study is not represented to scale. Dashed lines represent the limit of detection of the enzyme-linked immunosorbent assay, which varied from plate to plate, from 3·1 to 4·1 EU in the parent study (17) and from 5·5 to 7·4 EU in the extension study.

When considering anti-*S. sonnei* LPS IgG GMCs of participants in the boosted group across both the parent and extension trials, baseline antibody levels in the extension trial (GMC of 24 [95% CI: 4.12–145]) had decreased by ~17% compared to those at 6 months after the three-dose primary vaccination series (GMC of 29 [3.15–261]) (Supplementary Table 3). A significant individual anamnestic response was observed for each participant in the boosted group, including those primed with only 0.059/1 μg of 1790GAHB in the parent trial (Figure 5). Of note, two of the participants, primed with 0.059/1 μg or 0.29/5 μg formulations, respectively, had low anti-*S. sonnei* LPS IgG at 28 days after the third primary dose in the parent study and undetectable levels both at 6 months post-primary vaccination and at the time of the booster dose. These two participants showed post-booster antibody levels peaking at 94 and 282 EU, respectively, at 14 days post-boosting with 1790GAHB in the extension trial. One individual, for whom anti-*S. sonnei* LPS IgG of 1,099 was observed at 28 days post-third primary vaccination, maintained high antibody levels up to re-enrolment in the extension study (908 EU), which further increased following the booster dose and peaked at 14 days post-boosting (4465 EU).

### Safety

The most commonly reported solicited local AE was injection site pain after vaccination, reported by 86% of participants in either the boosted and vaccine-naïve groups. Erythema and induration were each reported by one participant in the boosted and the vaccine-naïve groups, respectively. Most of the local AEs were mild to moderate in severity, had the onset within 6 h post-vaccination and resolved within 4 days. Severe pain was reported by a single participant in the vaccine-naïve group (Table 2).

**Table 2:** Summary of local and systemic solicited adverse events (full analysis set).

**AE**	**Severity**	**Boosted group (*N* = 7)**	**Vaccine-naïve group (*N* = 28)**
**SOLICITED LOCAL ADVERSE EVENTS**, ***n*** **(%)**			
Pain	Any	6 (86)	24 (86)
	Severe	0	1 (4)
Erythema	Any	1 (14)	0
	Severe	0	0
Induration	Any	0	1 (4)
	Severe	0	0
**SOLICITED SYSTEMIC ADVERSE EVENTS**, ***n*** **(%)**			
Arthralgia	Any	1 (14)	3 (11)
	Severe	0	0
Chills	Any	1 (14)	1 (4)
	Severe	0	0
Fatigue	Any	3 (43)	11 (39)
	Severe	0	1 (4)
Headache	Any	1 (14)	5 (18)
	Severe	0	0
Malaise	Any	1 (14)	2 (7)
	Severe	0	0
Myalgia	Any	2 (29)	8 (29)
	Severe	0	0
Fever	≥38.0	0	0

*AE, adverse event; N, number of participants included in the analyses; n (%), number (percentage) of participants in each group. Severe solicited adverse events were defined as >100 mm (erythema, induration), or as preventing normal daily activities (pain, headache, arthralgia, chills, fatigue, malaise, myalgia)*.

The most commonly reported solicited systemic AEs were fatigue, myalgia, headache, and arthralgia, reported by ≥39, 29, ≥14, and ≥11% of participants in either group. Overall, no considerable differences were observed between the two groups in the percentage of participants reporting each of the systemic AEs. The majority of solicited systemic AEs were mild to moderate in severity, had onset within 6 h post-vaccination and were resolved within 4 days post-vaccination. None of the study participants experienced fever. One vaccine-naïve participant reported severe fatigue, 6 h after vaccination (Table 2). Three adults (43%) in the boosted group and five (18%) in the vaccine-naïve group were administered analgesics/antipyretics for the treatment of pain/fever occurring within seven days post-vaccination.

Unsolicited AEs were reported by four (57%) participants in the boosted group, compared with seven (25%) in the vaccine-naïve group. The most commonly reported categories of unsolicited AEs were classified by MedDRA system organ class as nervous system disorders (headache in two [29%] individuals in the boosted group and two [7%] participants in the naïve group), or musculoskeletal and connective tissue disorders (arthralgia and musculoskeletal pain in the boosted group and coccydynia in the vaccine-naïve group, each reported by one participant). All other classes of unsolicited AEs were reported by < 2 participants. All reported unsolicited AEs were mild or moderate in severity and were resolved at the time of study termination, with the exception of hemorrhoids in one participant in the vaccine-naïve group. One individual in the vaccine-naïve group reported an AESI (neutropenia), which was assessed by the investigator as at least possibly-related to the study vaccine. The episode was asymptomatic and mild in nature and was resolved by study end. No deaths or SAEs were reported in the study.

## Discussion

This study had some limitations due to the small sample size of the boosted group and to the different dose levels received by boosted volunteers in the primary vaccination; additionally, the immunological analyses, which were descriptive in nature, did not include functional assays or assessment of the cell-mediated immunity.

However, this was the first study to assess the longevity of the anti-*S. sonnei* LPS IgG response and the immunogenicity and safety of a booster dose of 1790GAHB, an investigational GMMA vaccine against *S. sonnei*.

A dose of 1790GAHB, administered to adults with anti-*S. sonnei* LPS antibody levels at or below the limit of detection before priming, induced a clear booster response 2–3 years after the completion of a three-dose primary series. A single dose of 1790GAHB administered to vaccine-naïve adults also elicited a robust increase in anti-*S. sonnei* LPS IgG. However, the response elicited by 1790GAHB was consistently higher in participants previously primed with three vaccine doses than in vaccine-naïve adults. A similar conclusion can be drawn by comparing the 1790GAHB responses at 28 days post-vaccination in the boosted group in the extension trial (GMC = 623 EU) with the response of the same participants after their first injection in the parent trial (GMC = 8.56 EU) or in participants with undetectable antibody at baseline and vaccinated with a high dose vaccine (GMC = 143 EU).

The use of alternate ways of assessing magnitude of antibody response (i.e., seroresponse rate and percentages of participants with ≥121 EU) was consistent with the observations made based on GMCs. A substantially higher response in the boosted group compared to the vaccine-naïve group was observed, indicating a clear booster response in previously vaccine-primed participants, even those receiving primary doses of OAg/protein content as low as 0.059/1 μg. The data also suggested that individuals with no pre-existing antibodies at the time of first vaccination can further benefit from the administration of a booster dose of 1790GAHB at 2–3 years post-primary vaccination.

It has been previously reported that there is a significant correlation between serotype-specific anti-LPS IgG antibodies in serum and resistance to shigellosis (20–22). Furthermore, episodes of *Shigella* diarrhea confer protection against future illness due to infection with the same, but not other serotypes (23). It is therefore likely that an effective public health vaccine will need to include antigens from most of the epidemiologically relevant *Shigella* serotypes and induce long-lived high specific anti-*Shigella* LPS antibody levels in immunologically-naïve individuals such as young children, in whom the burden of shigellosis is the highest. This study was conducted in adults with very low antibody levels prior to initial vaccination, emulating populations with no previous exposure to natural infection, and assessed long-term persistence of antibody levels. By evaluating volunteers from the parent trial with low pre-vaccination anti-LPS levels, we found that antibodies against *S. sonnei* persisted up to 3 years following primary vaccination with 1790GAHB and increased considerably after a booster dose. All participants with measurable antibodies at 6 months after the primary vaccination series still had substantial antibody levels at the time of boosting 2–3 years later. Three of them, including the individual with the highest antibody level of about 1,000 EU at 1 month post-third primary vaccination, maintained their antibody levels without considerable change throughout the entire duration of the studies. Over a period of ~3 years, between the 1 month post-primary and the pre-booster time points, there was a 2.4-fold decrease in the GMC. Most of this drop occurred in the 6 months following primary vaccination, with just a 1.2-fold drop over the remaining period. Although the comparison is limited by the small number of participants enrolled in this extension study, we observed antibody decay rates very different from those previously reported following vaccination with *Shigella* OAg-specific conjugate vaccine. In one study conducted among Israeli adults, who received a single vaccination of a conjugate vaccine composed of the O-specific polysaccharides of *S. sonnei* covalently bound to *Pseudomonas aeruginosa* recombinant exoprotein A (*S. sonnei*-rEPA), IgG levels declined 2.3-fold over a time period of 6 months after vaccination and another 2.2-fold over the next 18 months, compared with levels at 2 weeks post-vaccination (24). In a second Israeli adult study, antibody levels elicited by the same vaccine decayed 3.4-fold over 4 months post-vaccination in participants not infected with *S. sonnei* (25), while in a study in 4 to 7-year-old Israeli children, vaccinated twice with *S. sonnei*-rEPA, antibody levels declined 4.3-fold over 20 weeks following the second injection (26). Moreover, in our study, an additional vaccination with 1790GAHB elicited an anamnestic response in all participants of the boosted group, regardless of the OAg/protein content of 1790GAHB received during priming 2–3 years earlier.

The incidence of solicited AEs was similar between the boosted and vaccine-naive groups, showing that no increased reactogenicity is expected following a fourth administration of 1790GAHB vaccine at 2–3 years post-primary vaccination. As in previous studies assessing the reactogenicity and safety of the 1790GAHB vaccine, pain at injection site was the most common solicited AE (17, 18). A lower frequency of both local and systemic reactions was reported compared with that following the first dose with the same OAg/protein content of vaccine (1.5/25 μg) administered to Kenyan adults (18). Neutropenia was collected as an AESI due to the occurrence of such episodes in previous studies, including the parent trial (17), but only one mild and asymptomatic neutropenia episode was reported in the current extension study, in a previously unprimed participant. Overall, the safety results of this trial confirmed the acceptable safety profile of 1790GAHB shown in previous clinical trials (17, 18).

## Conclusions

A single administration of the 1790GAHB vaccine elicited a booster response in healthy European adults receiving a three-dose primary schedule 2–3 years earlier and having undetectable anti-*S. sonnei* LPS IgG prior to primary vaccination. A strong immune response was also induced in vaccine-naïve participants. 1790GAHB was well tolerated in all vaccine-naïve study participants, with no increased reactogenicity observed in boosted individuals. These results support further studies investigating the administration of GMMA-based *Shigella* vaccine using primary and booster vaccination schedules in adults and children. As cross protection against other *Shigella* serotypes is unlikely for this monovalent *S. sonnei* vaccine, further development will be based on a multicomponent vaccine including GMMA from other epidemiologically relevant *Shigella* serotypes.

## Data Sharing

Anonymized individual participant data and study documents can be requested for further research from www.clinicalstudydatarequest.com.

## Author Contributions

AS, LM, AP, JA, ASS, EM, PL, AN, OL, NN, PF, and AL were involved in the study conception and design. OL, AS, LM, AP, EM, PL, AN, LS, NN, VC, and AL were involved in acquisition and generation of data. OL, AP, EM, PL, NN, ASS, VC, and AL performed the study. OL, AS, LM, AP, ASS, PL, AN, VC, and PF were involved in data analysis and data interpretation. All authors contributed substantially to the development of the manuscript and approved the final version.

### Conflict of Interest Statement

AN, VC, ASS, PF, NN, LS, JA, EM, AS, LM, and AP are employees of the GSK group of companies. OL received grants from GSK to conduct the study and reports personal fees from Innavirvax, Sanofi Pasteur, Pfizer, and Janssen for consultancy, outside the submitted work. AS, LM, and AP report grants from European Union Seventh Framework Programme Grants “ADITEC” and STPENTERICS, during the conduct of the study, but outside the submitted work. AS has a patent WO16202872 pending to GlaxoSmithKline Biologicals SA, a patent US2016289632 pending to GlaxoSmithKline Biologicals SA, and a patent US2015202274 pending to GlaxoSmithKline Biologicals SA. LM reports grants from Bill & Melinda Gates Foundation, outside the submitted work. In addition, LM has a patent WO2016202872 issued. AN and PF report grants from BMGF, outside the submitted work. The remaining authors declare that the research was conducted in the absence of any commercial or financial relationships that could be construed as a potential conflict of interest.
