# Supplementary material for: Booster Vaccination With GVGH Shigella sonnei 1790GAHB GMMA Vaccine Compared to Single Vaccination in Unvaccinated Healthy European Adults: Results From a Phase 1 Clinical Trial
Source: Front Immunol. 2019 Mar 8;10:335. doi: 10.3389/fimmu.2019.00335 (PMC6418009; doi:10.3389/fimmu.2019.00335)
Supplement: Supplementary file 1 [file Data_Sheet_1.docx]

# *Supplementary material*

## 1 Supplementary text 1. Inclusion/exclusion criteria

Eligible individuals were:

- Males and females, aged 22–50 years, who were previously vaccinated, with either the 1790GAHB vaccine (3 doses) or placebo, in the parent study (NCT02017899) and who had undetectable antibody titers at baseline, or males and females, aged 22–50 years, who were not part of the parent study.
- Individuals who, after the nature of the study has been explained to them, and prior to any protocol specific procedures being performed, have given written consent according to local regulatory requirements.
- Individuals in good health as determined by the outcome of medical history, physical examination, hematological blood tests and clinical judgment of the investigator.
- For women of child-bearing potential, a negative urinary pregnancy test prior study vaccination and willingness to use acceptable birth control measures for the entire study duration was required.
- Individuals affiliated to a social security regimen.

The following individuals were excluded from the study:

- Individuals with behavioral or cognitive impairment or psychiatric disease that, in the opinion of the investigator, may interfere with the subject's ability to participate in the study.
- Individuals with any progressive or severe neurological disorder, seizure disorder or Guillain-Barré syndrome.
- Individuals who were not able to understand and to follow all required study procedures for the whole period of the study.
- Individuals with known hepatitis B or C or suspected HIV infection or HIV related disease with history of an autoimmune disorder or any other known or suspected impairment/alteration of the immune system.
- Individuals with progressive, unstable or uncontrolled clinical conditions.
- Individuals with hypersensitivity, including allergy, to any component of vaccines, medicinal products or medical equipment for which use is foreseen in this study.
- Individuals with a known bleeding diathesis, or any condition that may be associated with a prolonged bleeding time.
- Individuals with clinical conditions representing a contraindication to intramuscular vaccination and blood draws.
- Individuals with abnormal function of the immune system resulting from: clinical conditions; systemic administration of corticosteroids for more than 14 consecutive days within 90 days prior to informed consent; administration of antineoplastic and immunomodulating agents or radiotherapy within 90 days prior to informed consent.
- Individuals with who received immunoglobulins or any blood products within 180 days prior to informed consent.
- Study personnel as an immediate family or household member.
- Individuals with any other clinical condition that, in the opinion of the investigator, might interfere with the results of the study or pose additional risk due to participation in the study.
- Individuals who have received an investigational product in another clinical trial 28 days prior to first study visit or intended to receive another investigational product at any time during the conduct of this study.
- Individuals who received any other vaccines within 4 weeks prior to enrollment in this study or who were planning to receive any vaccine within the entire study duration. Inactivated influenza vaccine was allowed, but only 4 weeks earlier or 4 weeks later than the date of immunization.
- Individuals who have received blood, blood products, and/or plasma derivatives including parenteral immunoglobulin preparations in the past 180 days.
- Individuals with body temperature >38.0℃ within 3 days of intended study vaccination.
- Individuals with a body mass index >30 kg/m^2^.
- Individuals with history of substance or alcohol abuse within the past 2 years.
- Women who were pregnant or breast-feeding, or were of childbearing age who have not used or did not plan to use acceptable birth control measures, for the duration of the study.
- Females with history of stillbirth, neonatal loss, or previous infant with anomaly.
- Individuals who had a previously laboratory confirmed or suspected disease caused by *Shigella sonnei*.
- Individuals who have had household contact with/and or intimate exposure to an individual with laboratory confirmed *S. sonnei*.

## 2 Supplementary Tables.

## Supplementary table 1. Anti-*S. sonnei* LPS IgG geometric mean concentrations and geometric mean ratios, by time point (full analysis set for immunogenicity)

|  | **GMC [EU] (95% CI)** | |  |  | **GMR (95% CI)** | |
| --- | --- | --- | --- | --- | --- | --- |
|  | **Boosted group** | **Vaccine-naïve group** |  | **Ratio** | **Boosted group** | **Vaccine-naïve group** |
| D1 | 24 (4·12–145) | 14 (8·34–23) |  |  | - | - |
| D8 | 168 (32–889) | 32 (17–61) |  | D8/D1 | 6·87 (2·69–18) | 2·24 (1·42–3·55) |
| D15 | 883 (249–3126) | 97 (51–187) |  | D15/D1 | 36 (14–93) | 7·09 (4·06–12) |
| D29 | 623 (159–2446) | 100 (54–187) |  | D29/D1 | 26 (8·88–73) | 7·40 (4·34–13) |
| D85 | 451 (113–1797) | 89 (48–166) |  | D85/D1 | 18 (5·37–64) | 6·47 (3·78–11) |

LPS, lipopolysaccharide; IgG, immunoglobulin G; GMC, geometric mean concentration; EU, enzyme-linked immunosorbent assay units; CI, confidence interval; GMR, geometric mean ratio; D, day.

**Note:** D1, D8, D15, D29, and D854 correspond to vaccination day at 7, 14, 28, and 84 days post-vaccination.

## Supplementary table 2. Percentage of participants with seroresponse and anti-*S. sonnei* LPS IgG ≥121 EU, by time point (full analysis set for immunogenicity)

|  | **Seroresponse (95% CI)** | |  | **% with anti-*S. sonnei* LPS IgG ≥121 EU (95% CI)** | |
| --- | --- | --- | --- | --- | --- |
|  | **Boosted group** | **Vaccine-naïve group** |  | **Boosted group** | **Vaccine-naïve group** |
| D1 | - | - |  | 14 (0·36–57·9) | 7 (0·9–24·3) |
| D8 | 86 (42·1–99·64) | 24 (9·4–45·1) |  | 71 (29·0–96·3) | 28 (12·1–49·4) |
| D15 | 100 (59·0–100) | 70 (49·8–86·2) |  | 86 (42·1–99·64) | 37 (19·4–57·6) |
| D29 | 100 (59·0–100) | 69 (48·2–85·7) |  | 86 (42·1–99·64) | 38 (20·2–59·4) |
| D85 | 100 (59·0–100) | 67 (46·0–83·5) |  | 86 (42·1–99·64) | 37 (19·4–57·6) |

LPS, lipopolysaccharide; IgG, immunoglobulin G; EU, enzyme-linked immunosorbent assay units; CI, confidence interval; D, day.

Note: Seroresponse to vaccination was defined as an increase in the anti-*S. sonnei* LPS serum IgG level of ≥50% for participants with baseline (D1) levels >50 EU or an increase of ≥25 EU for participants with pre-vaccination (D1) levels ≤50 EU.

D1, D8, D15, D29 and D85 correspond to vaccination day and 7, 14, 28 and 84 days post-vaccination.

## Supplementary table 3. Anti-*S. sonnei* LPS IgG geometric mean concentrations and geometric mean ratios for participants in the boosted group, by doses received and time point in the parent study

|  | **GMC [EU] (95% CI)** | | | |
| --- | --- | --- | --- | --- |
|  | **0·059/1 µg (N=4)** | **0·29/5 µg (N=1)** | **2·9/50 µg (N=2)** | **Total (N=7)** |
| D1 | 2·57 (2·57–2·57) | 2·57 | 3·10 (0·015–624) | 2·71 (2·13–3·44) |
| D29 | 2·57 (2·57–2·57) | 2·46 | 178 (1·22*10^-9^–7·2580700269) | 8·56 (1·12–66) |
| D57 | 18 (3·69–84) | 4·50 | 209 (0·0047–9269102·6954) | 29 (6·32–137) |
| D85 | 36 (2·81–464) | 5·50 | 489 (0·017–14342611·555) | 58 (8·94–378) |
| D225 | 17 (0·69–429) | 1·85 | 313 (0·0012–83193932·62) | 29 (3·15–261) |

LPS, lipopolysaccharide; IgG, immunoglobulin G; N, number of participants; GMC, geometric mean concentration; EU, enzyme-linked immunosorbent assay units; CI, confidence interval; D, day.

Note: Primary doses were administered at D1, D29 and
